# CUDA-Accelerated Geodesic Ray-Tracing for Fiber Tracking

**DOI:** 10.1155/2011/698908

**Published:** 2011-09-20

**Authors:** Evert van Aart, Neda Sepasian, Andrei Jalba, Anna Vilanova

**Affiliations:** ^1^Department of Mathematics and Computer Science, Eindhoven University of Technology, 5600 MB Eindhove, The Netherlands; ^2^Department of Biomedical Engineering, Eindhoven University of Technology, 5600 MB Eindhoven, The Netherlands

## Abstract

Diffusion Tensor Imaging (DTI) allows to noninvasively measure the diffusion of water in fibrous tissue. By reconstructing the fibers from DTI data using a fiber-tracking algorithm, we can deduce the structure of the tissue. In this paper, we outline an approach to accelerating such a fiber-tracking algorithm using a Graphics Processing Unit (GPU). This algorithm, which is based on the calculation of geodesics, has shown promising results for both synthetic and real data, but is limited in its applicability by its high computational requirements. We present a solution which uses the parallelism offered by modern GPUs, in combination with the CUDA platform by NVIDIA, to significantly reduce the execution time of the fiber-tracking algorithm. Compared to a multithreaded CPU implementation of the same algorithm, our GPU mapping achieves a speedup factor of up to 40 times.

## 1. Introduction

Diffusion-Weighted Imaging (DWI) is a recent, noninvasive Magnetic Resonance Imaging (MRI) technique that allows the user to measure the diffusion of water molecules in a given direction. Diffusion Tensor Imaging (DTI) [1] describes the diffusion measured with DWI as a second-order tensor. DWI works on the knowledge that the diffusion of water molecules within biological tissue is influenced by the microscopic structure of the tissue. The theory of Brownian motion dictates that molecules within a uniform volume of water will diffuse randomly in all directions, that is, the diffusion is *isotropic*. However, in the presence of objects that hinder the diffusion of water in some specific directions, the diffusion will become *anisotropic*. In fibrous tissue, the diffusion of water will be large in the direction parallel to the fibers and small in perpendicular directions. Therefore, DWI data is used to deduce and analyze the structure of fibrous tissue, such as the white matter of the brain, and muscular tissue in the heart. DWI data has been used during the planning stages of neurosurgery [2], and in the diagnosis and treatment of certain diseases, such as Alzheimer's disease [3], multiple sclerosis [4], and strokes [5]. Since the tissue of the white matter is macroscopically homogeneous, other imaging techniques, such as T2-weighted MRI, are unable to detect the structure of the underlying fibers, making DWI uniquely suitable for in vivo inspection of white matter.

The process of using the measured diffusion to reconstruct the underlying fiber structure is called fiber tracking. Many different fiber tracking algorithms have been developed since the introduction of DTI. This paper focuses on an approach in which fibers are constructed by finding geodesics on a Riemannian manifold defined by the DTI data. This technique, called geodesic ray-tracing [6, 7], has several advantages over other ones, such as its relatively low sensitivity to measurement noise, and its ability to identify multiple solutions between two points, which makes it suitable for analysis of complex structures.

One of the largest downsides of this algorithm is that it is computationally expensive. Our goal is to overcome this problem by mapping the geodesic ray-tracing algorithm onto the highly parallel architecture of a Graphical Processing Unit (GPU), using the CUDA programming language. Since fibers can be computed independently of each other, the geodesic ray-tracing algorithm can be meaningfully parallelized. As a result, the running time can be reduced by a factor of up to 40, compared to a multithreaded CPU implementation. The paper describes the structure of the CUDA implementation, as well as the relevant design considerations.

In the next section, we discuss the background theory related to our method, including DTI and the geodesic ray-tracing algorithm. Next, we give an overview of past research related to the GPU-based acceleration of fiber tracking algorithms. In Section 4, the implementation of the geodesic ray-tracing algorithm on a GPU using CUDA is discussed. Next, we show benchmarking results and optimization strategies for the CUDA implementation in Section 5, followed by a discussion of the results in Section 6, and a conclusion in Section 7.

## 2. Background

### 2.1. Diffusion Tensor Imaging

**Figure 1:**
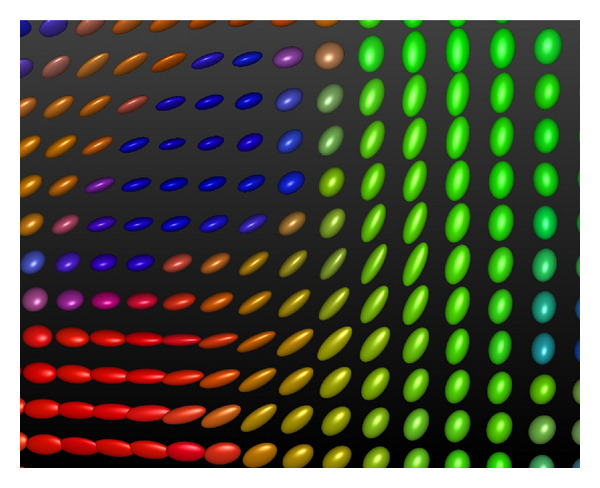
3D glyphs visualizing diffusion tensors. The orientation and sharpness of the glyphs depend on the eigenvectors and eigenvalues of the diffusion tensor, respectively. In this image, the glyphs have been colored according to the orientation of the main eigenvector (e.g., a main eigenvector of (1,0, 0) corresponds to a red glyph, while a main eigenvector of (0,0, 1) corresponds to a blue glyph). This image was generated in the DTITool [11].

DTI allows us to reconstruct the connectivity of the white matter, thus giving us greater insight into the structure of the brain. After performing DWI for multiple different directions, we can model the diffusion process using a second-order tensor **D** [1, 8]. **D** is a 3  ×  3 positive-definite tensor, which can be visualized as an ellipsoid defined by its eigenvectors and eigenvalues, as shown in Figure 1. Using the eigenvalues of a tensor, we can quantify its level of anisotropy using *anisotropy measures* [9, 10]. In areas with nearly isotropic diffusion, tensor ellipsoids will be nearly spherical, and anisotropy measure values will be low, while in areas with highly anisotropic diffusion (due to the presence of fibers), ellipsoids will be sharp and elongated, and anisotropy values will be high. In anisotropic areas, the eigenvector corresponding to the largest eigenvalue (the *main eigenvector*) will indicate the direction of the fibrous structure.

### 2.2. Fiber Tracking Algorithms

**Figure 2:**
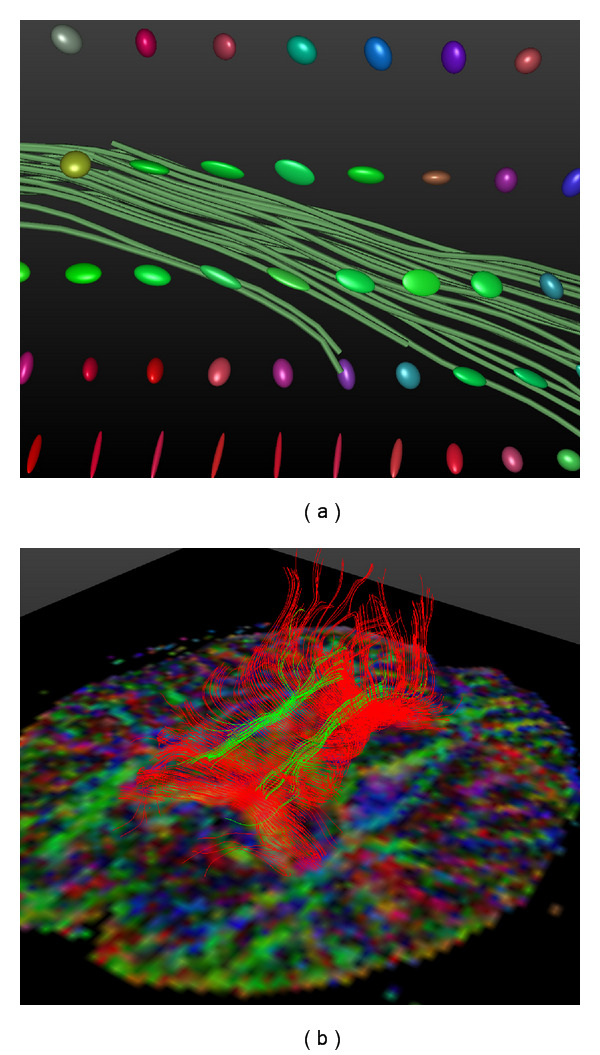
(a) Small group of fibers generated using a simple streamline method. The main eigenvectors of the diffusion tensors determine the local orientation of the fibers. (b) Fibers showing part of the Cingulum and the corpus callosum. Both the glyphs in the left image and the plane in the right image use coloring based on the direction of the main eigenvector, similar to Figure 1. Both images were generated in the DTITool [11].

*DTI Fiber Tracking* is the process of digitally reconstructing the pathways of fibers in fibrous tissue using the DTI tensor data, with the aim of deducing the structure of the tissue. A common approach to fiber tracking is to track lines from one or more seed points, using a set of differential equations. The most straightforward fiber tracking algorithm (generally called the *streamline* method) uses the direction of the main eigenvector as the local orientation of the fibers [12]. Thus, a fiber in biological tissue may be reconstructed by integration of the main eigenvector, using an Ordinary Differential Equation (ODE) solver such as Euler's method.  Figure 2 illustrates the relation between the diffusion tensors and the resulting fiber trajectories. Fiber tracking algorithms based on this approach have been shown to achieve acceptable results [13, 14], but are limited in their accuracy by a high sensitivity to noise and to the partial volume effect [15, 16].

One possible solution to the limitations of classic streamline methods is to use a global minimization solution, for example, a *front-propagation method*. In such a method, a front is propagated from a seed point throughout the entire volume [17–19]. The local propagation speed of the front depends on the characteristics of the DTI image, and fibers are constructed by back-tracing through the characteristics of the front. A subset of these front-propagation methods use the theory of geodesics to find potential fiber connections [19–21]. These algorithms generally compute the geodesics (defined as the shortest path through a tensor-warped space) by solving the stationary Hamilton-Jacobi (HJ) equation. Geodesic algorithms have been shown to produce good results and are generally more robust to noise than simple streamline methods. One disadvantage, however, is that they generally only find a single possible connection between target regions, which is often not the correct solution in very complex areas. Furthermore, research has shown it is possible to have multiple fiber connections between regions of the white matter [22].

### 2.3. Geodesic Ray-Tracing

**Figure 3:**
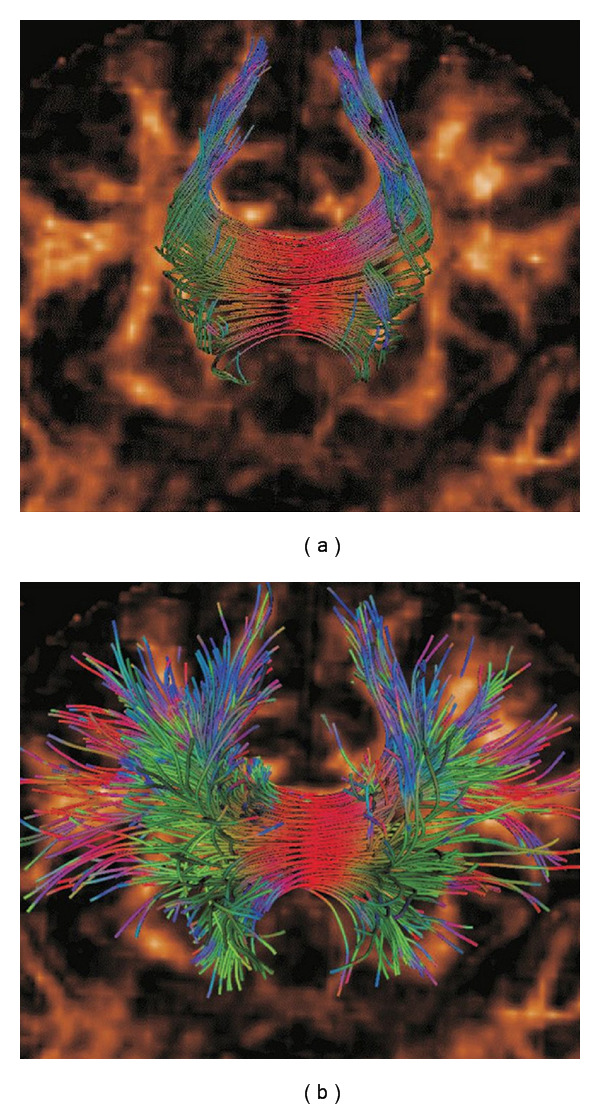
Fibers of part of the corpus callosum, computed using the streamlines method (a) and the geodesic ray-tracing method (b). Fibers were computed from seed points located in the center of the corpus callosum and are colored according to their local orientation (similar to the glyphs in Figure 1). Unlike the streamline method, which only captures the most dominant bundles of the corpus callosum, the geodesic ray-tracing method is able to correctly detect the divergence of the fiber bundles. This image was generated in the DTITool [11].

The focus of this paper is a relatively new fiber tracking algorithm based on geodesics, as proposed by Sepasian et al. [6, 7]. The main advantages of this algorithm, compared to those discussed in the previous section, are the fact that it provides a *multivalued* solution (i.e., it allows us to find multiple geodesics between regions in the brain), and the fact that it is able to detect fibers in regions with low anisotropy (e.g., regions of the white matter with crossing fiber bundles). In Figure 3, we show that this algorithm is capable of detecting complex structural features, such as the divergence of the corpus callosum, which cannot be captured using streamline-based methods. Detailed validation of this algorithm is considered beyond the scope of this paper; for an in-depth discussion on the validity and applicability of the algorithm, we refer the reader to the works by Sepasian et al. [6, 7].

A geodesic is defined as the shortest path on a Riemannian manifold. This is a real, differentiable manifold, on which each tangent space is equipped with a so-called *Riemannian metric*, which is a positive-definite tensor. Roughly speaking, the elements of the metric tensor are an indication of the *cost* of (or *energy* required for) moving in a specific direction. For DTI data, an intuitive choice for the metric is the *inverse* of the diffusion tensor. Large values in the DTI tensor correspond to small values in its inverse, indicating low diffusion costs, and vice versa. Locally, a geodesic will tend to follow the direction with the lowest metric value, which is analogous to the direction with the highest diffusion. We define the Riemannian metric as *G* = *D*^−1^, where *D* is the diffusion tensor.

In the algorithm discussed in this paper, the trajectory of a fiber is computed iteratively by numerically solving a set of ODEs. The ODEs used to compute the trajectory of the fiber are derived from the theory of geodesics in a Riemannian manifold, as shown below.

Let **x**(*τ*) be a smooth, differentiable curve through a volume described by parameter *τ* = [0, *T*], with derivative vector $\dot{x}(\tau )$. We define the Riemannian length of **x**(*τ*) as follows:

(1) $$\begin{array}{c} L\left(x\right)=\overset{T}{\underset{0}{\int }}\sqrt{{\dot{x}}^{T}G\dot{x}}d\tau \end{array}.$$

The geodesic is the line that *minimizes* the geodesic length of (1). We can use the Euler-Lagrange equations to translate this function to a set of ODEs, as described in detail by Jost [23].

Let ${\dot{x}}^{\gamma }$ and ${\ddot{x}}^{\gamma }$ be the first and second derivatives with regard to *τ*, respectively, of the geodesic for dimension *γ* = (1,  2,  3). The ODEs that allow us to compute the geodesics are given by the following equation:

(2) $${\ddot{x}}^{\gamma }+\overset{3}{\underset{\alpha =1}{\sum }}\;\overset{3}{\underset{\beta =1}{\sum }}\Gamma_{\alpha \beta }^{\gamma }{\dot{x}}^{\alpha }{\dot{x}}^{\beta }=0,$$

where Γ_*αβ*_^*γ*^ are the so-called *Christoffel symbols*, defined as follows:

(3) $$\Gamma_{\alpha \beta }^{\gamma }=\overset{3}{\underset{\sigma =1}{\sum }}\frac{1}{2}\left[g^{\gamma \sigma }\left(\frac{\partial }{\partial x_{\alpha }}g_{\beta \sigma }+\frac{\partial }{\partial x_{\beta }}g_{\alpha \sigma }-\frac{\partial }{\partial x_{\sigma }}g_{\alpha \beta }\right)\right].$$

Here, *g*_*ij*_ represents element (*i*, *j*) of the inverse diffusion tensor, while *g*^*ij*^ represents an element of the *original* diffusion tensor. We note that, in order to compute all Christoffel symbols, we need to compute the derivatives of the inverse DTI tensor in all three dimensions.

Given an initial position and an initial direction, we can construct a path through the 3D DTI image, using the second-order Runge-Kutta ODE solver. The initial position is usually specified by the user, who is interested in a particular area of the tissue (in our case, the white matter). For the initial direction, we can use a large number of directions per seed points (distributed either uniformly on a sphere, or around the main eigenvector), and see which of the resulting fibers intersect some user-specified target region. Doing so increases our chances of finding all valid connections between the seed point(s) and the target region(s).

This approach, which is referred to as the Region-to-Region Connectivity approach, requires a suitable *Connectivity Measure* [24], which quantifies the strength of the connection between seed point and target region. In other words, this measure symbolizes the probability that a computed geodesic corresponds to an actual fibrous connection in the white matter. While this paper does not discuss the implementation of the Region-to-Region Connectivity approach, we do note that in order to reliably find all geodesics between the seed point(s) and the target region, we need to compute a large amount of trajectories in all directions. This need for a large amount of fibers, in combination with the high computational complexity of the algorithm itself, motivates our decision to parallelize the geodesic ray-tracing algorithm.

## 3. Related Work

The possibility of using the GPU to accelerate fiber tracking algorithms (using CUDA or other languages) has recently been explored in other literature [25–28]. These implementations use either geometric shaders or fragment shaders to accelerate the streamline tracking algorithm. With the exception of Mittmann et al. [28], who introduce a stochastic element, these papers all use the simple streamline method for fiber tracking, in which the main eigenvector of the DTI tensor is used as the direction vector for the integration step. In addition to using algorithms with a lower computational complexity than the geodesic ray-tracing algorithm discussed in Section 2.3, these implementations differ from ours in the sense that they use GPU shaders to compute the fibers, while we use CUDA, which offers higher flexibility and a more gentle learning curve than programmable GPU shaders [29].

More recently, Mittmann et al. introduced a GPU implementation of a simple streamline algorithm using CUDA [30]. Compared to a multithreaded CPU implementation, this GPU implementation allows for a significantly higher frame rate, enabling real-time, interactive exploration of large groups of fibers. The speedup factor, based on the number of frames per second, is between 10 and 20 times. The paper's main focus, however, is interactivity, and a technical discussion of the advantages and limitations of CUDA in the context of fiber tracking algorithms is omitted.

Jeong et al. [31] have developed a CUDA implementation of a fiber tracking algorithm based on the Hamilton-Jacobi equation, which computes the fiber pathways by propagating a front throughout the entire volume. Their solution parallelizes the propagation of the front by dividing the DTI image into blocks of 43 voxels, after which the front is propagated through a number of such blocks in parallel. This approach has been shown to be 50 to 100 times faster than sequential implementations of similar algorithms on a CPU. However, as stated in Section 2.3, the HJ algorithm on which they base their implementation is not able to find multiple connections between target regions. Furthermore, the front-propagation algorithm used by Jeong et al. requires a fundamentally different parallelization approach from our ray-tracing method.

We therefore conclude that our solution is the first CUDA-aided acceleration of a fiber tracking algorithm of this complexity. As will be shown in the next sections, the complex nature of the algorithm introduces a number of challenges to the parallelization process. Furthermore, the advantages of the geodesic ray-tracing algorithm, as discussed in Section 2.3, suggest that its implementation will also have practical applications.

## 4. Geodesic Fiber Tracking on the GPU Using CUDA

### 4.1. Algorithm Overview

In Section 2.3, we introduced a system of ODEs which can be used to compute the trajectory of a fiber, given an initial position and direction. The basis of our algorithm is an ODE solver which numerically solves (2) using a fixed integration step length. Specifically, let *x*_*i*_^*γ*^ be the coordinate of point *i* along the fiber for dimension *γ*, and let ${\dot{x}}_{i}^{\gamma }$ be the local direction of the fiber in this point. Using Euler as the ODE solver, we can compute *x*_*i*+1_^*γ*^ and ${\dot{x}}_{i+1}^{\gamma }$ (i.e., the position and direction at the next time step) as follows:

(4) $$\begin{array}{c} \begin{array}{c} x_{i+1}^{\gamma }=x_{i}^{\gamma }+h{\dot{x}}_{i}^{\gamma }, \end{array}\\ {\dot{x}}_{i+1}^{\gamma }={\dot{x}}_{i}^{\gamma }-h\overset{3}{\underset{\alpha =1}{\sum }}\;\overset{3}{\underset{\beta =1}{\sum }}\Gamma_{\alpha \beta }^{\gamma }{\dot{x}}^{\alpha }{\dot{x}}^{\beta }. \end{array}$$

Here, *h* is a fixed step size, and Γ_*αβ*_^*γ*^ is the Christoffel symbol as defined in (3). In the implementation described below, we use a second-order Runge-Kutta ODE solver instead of the Euler solver, but the basic method of numerically solving the system of ODEs remains the same. When computing the Christoffel symbols, the four required tensors (inverse DTI tensors and its three derivates) are interpolated using trilinear interpolation.

To summarize, a single integration step of the ODE solver consists of the following actions.

(1) Compute the inverse diffusion tensor and its derivative in all three dimensions for the eight voxels surrounding the current fiber point (*x*_*i*_^*γ*^).
(2) Interpolate the four tensors at the current fiber point.
(3) Compute all Christoffel symbols. According to (2) and (3), we require nine symbols per dimension, for a total of 27 symbols. However, using the symmetric qualities of the diffusion tensor (and therefore, of its inverse and the derivates of its inverse), we can reduce this to 18 unique symbols.
(4) Using the Christoffel symbols, compute the position and direction of the next fiber point.
(5) Repeat steps 1 through 4 until some stopping condition is met. By default, the only stopping condition is the fiber leaving the volume of the DTI image.

**Figure 4:**
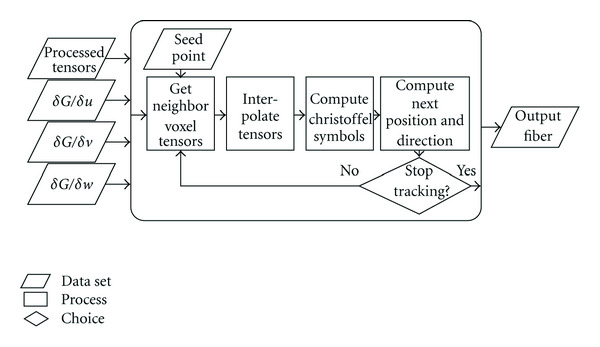
Flowchart for the geodesic fiber tracking algorithm. Using the four input tensor fields, we compute the trajectory of a fiber using a numerical ODE solver.

We note that the first step may be performed as a preprocessing step. While this increases the amount of memory required by the algorithm, it also significantly decreases the number of required computations per integration step. This pre-processing step has been implemented in CUDA, but due to its trivial implementation and relatively low running time (compared the that of steps 2 through 5), we do not discuss it in detail, instead focusing on the actual tracking process.  Figure 4 summarizes the tracking process of steps 2 through 5, assuming the four input tensor fields have been computed in a pre-processing step.

### 4.2. CUDA Overview

The Region-to-Region Connectivity approach outlined in Section 2.3 presents us with one significant problem: it requires the computation of a large number of geodesics. In combination with the relatively complex ODEs presented in (2) (compared to the ODEs used for simpler fiber tracking methods), this makes this approach computationally expensive. Our aim is to overcome this hurdle by implementing the algorithm in CUDA, of which we give a quick overview in this section.

NVIDIA's *Compute Unified Device Architecture* (CUDA) [32] is a way to facilitate General-Purpose computing on Graphics Processing Units (GPGPU). Modern GPUs contain large number of generic processors in parallel, and CUDA allows a programmer to utilize this large computational power for nongraphical purposes. In CUDA, a *kernel* (usually a small, simple function) is executed in parallel by a large number of *threads*, each working on one part of the input data. In a typical execution pattern, the host PC first uploads the input data to the GPU, then launches a number of threads that execute the kernel. The resulting data is then either downloaded back to the host PC or drawn on the screen.

A CUDA-enabled GPU generally consists of the *device memory*, which is between 512 MB and 2 GB on most modern GPUs, and a number of *multiprocessors* [33]. Each multiprocessor contains eight *scalar processors* (in most current-generation GPUs; newer generations will have more scalar processors per multiprocessor); a *register file*; a *shared memory* block, which enables communication between the scalar processors; and an *instruction unit*, which dispatches instructions to the processors. The type of parallelism in these multiprocessors is called *Single Instruction, Multiple Threads* (SIMT), which differs from Single Instruction, Multiple Data (SIMD) in the sense that the threads have some level of independence. Ideally, all active threads in a multiprocessor will execute the same instruction at the same time; however, unlike SIMD, SIMT also allows for branching threads using if-statements. While this does allow the programmer to create more complex kernels, it also adds an overhead in terms of execution time, since the different branches must be executed sequentially. Therefore, it is best to avoid branching behavior in kernels where possible.

One other important consideration when designing CUDA algorithms is the memory hierarchy. The two memory blocks local to each multiprocessor—the register file and the shared memory—both have low memory access latencies. However, the available space in these two memory blocks is limited, with typical values of 16 kB for the shared memory, and 16 or 32 kB for the register file. The device memory has far more storage space, but accessing this memory adds a latency of between 400 and 900 clock cycles [34]. The device memory contains four different memory spaces:

- *Constant Memory,* a small, read-only block best used for constant values;
- *Texture Memory,* a read-only space optimized for texture reads;
- *Global Memory,* the main random-access memory space;
- *Local Memory,* a per-thread extension of the register file.

**Figure 5:**
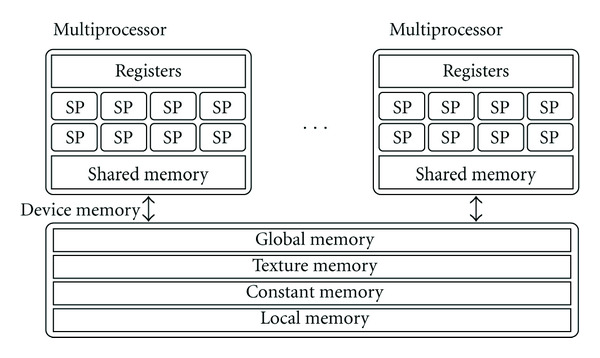
General structure of a CUDA-enabled GPU, showing the device memory, the multiprocessors, the scalar processors (SP), and the relevant memory spaces.

Local memory is used when the register file of a multiprocessor cannot contain all variables of a kernel. Since access latencies to the device memory are very high, the use of large kernels is generally best avoided. As a general rule, communication between the device memory and the multiprocessors should be kept as low as possible. A second important guideline is that the size of the kernels, in terms of the number of registers per thread and the amount of shared memory used per thread block, should be kept small. Doing so will allow the GPU to run more threads in parallel, thus increasing the *occupancy* of the scalar processors (i.e., the percentage of time that each scalar processor is active). The structure of a CUDA-enabled GPU is illustrated in Figure 5.

### 4.3. CUDA Implementation

Keeping in mind the advantages and limitations of CUDA as discussed in Section 4.2, we can design a CUDA implementation for the algorithm introduced in Section 2.3. As mentioned in step 3 of the algorithm as outlined in Section 4.1, we require the derivatives of the inverse of the DTI tensor in all three dimensions. We intuitively see that computing these derivatives for each point along the fiber would be far too costly in terms of the number of instructions, as computing these derivates for all eight surrounding voxels (using two-sided numerical derivation) would require the inversion of 32 diffusion tensors. Therefore, we decide to precompute them instead.

This gives us four input tensors per voxel: the diffusion tensor and the three derivatives of its inverse. With six unique elements per tensor and four bytes per value, this gives us a memory requirement of 4∗6∗4 = 96 Bytes per voxel. The algorithm also has the seed points as input, which contain the initial position and direction for each fiber. These seed points are determined by the user, for example, by specifying a region of interest in the image.

The output of the algorithm consists of a number of fibers, each consisting of a list of 3D coordinates. In postprocessing steps, these fibers can be filtered through the target region(s) and sorted according to their Connectivity Measure value, but these steps are not part of the CUDA implementation discussed herein. Since CUDA does not support dynamic memory allocation, we hard-coded a limit in the number of iteration steps for each fiber, and statically allocated an output array per fiber of corresponding size. We note here that the choice of this limit may impact performance: for high limits, more fibers will terminate prematurely (due to leaving the volume), leading to lower occupancy in later stages of the algorithm, while for lower limits, the start-up overhead of the CUDA algorithm may become relevant.

We intuitively recognize two different approaches for parallelization of the geodesic ray-tracing algorithm: *per-region* parallelization and *per-fiber* parallelization. The per-region approach would entail loading a small region of the image into the shared memory of a multiprocessor, tracking a number of fibers through this region (using one thread per fiber), and then loading the next region. While this approach would guarantee low memory bandwidth requirements between the multiprocessors and the device memory, it is impractical due to two reasons. First, it is impossible to guarantee that a region will contain a sufficient number of fibers to enable meaningful parallelization. Second, due to the limited size of the shared memory, these regions would be very small (no more than approximately 160 voxels per region), which would defeat the purpose of this approach. In other words, this approach requires some degree of *spatial coherence* between the fibers, and since we do not know their trajectories beforehand, ensuring this spatial coherence is highly impractical.

We therefore use the per-fiber parallelization approach, in which each thread computes a single fiber. The main advantage of this approach is that it does not require any spatial coherence between the fibers to efficiently utilize the parallelism offered by the GPU. As long as we have a high number of seed points and initial directions, all scalar processors of the GPU will be able to run their own threads individual of the other threads, thus minimizing the need for elaborate synchronization between threads, and guaranteeing a stable computational throughput for all active processors. The main disadvantage of this approach is that it requires a higher memory throughput than the per-region approach, as it does not allow us to avoid redundant memory reads.

The kernel that executes the fiber tracking process executes the following steps, as summarized in Figure 4:

(1) Fetch the initial position and direction of the fiber.
(2) For the current fiber position, fetch the diffusion tensor and the derivatives of its inverse, using trilinear interpolation.
(3) Using these four tensors, compute the Christoffel symbols, as defined in (3).
(4) Using the symbols and the current direction, compute the next position and direction of the fiber, using the ODEs of (2) with a second-order Runge-Kutta step.
(5) Write the new position to the global memory.
(6) Stop if the fiber has left the volume *or* the maximum number of steps has been reached. Otherwise, return to Step 2.

### 4.4. Using Texture Memory

As noted in the previous section, the main disadvantage of the per-fiber parallelization approach is that it does not allow us to avoid redundant reads. We can partially solve this problem by storing the input images in *texture memory*, rather than in global memory. Unlike global memory reads, texture memory reads are cached through a number of small caches. These caches can contain about 8 kB per multiprocessor, although the actual amount varies per GPU. While we argue that cached memory reads could reduce the required memory throughput, we note that the lack of spatial coherence between the fibers, coupled with the small cache sizes, will largely negate the positive effects of cached memory reads.

A second, more important advantage of the texture memory is the built-in texture filtering functionality. When reading texture data from a position located between grid points, CUDA will apply either nearest-neighbor or linear interpolation using dedicated texture filtering hardware. When storing the input data in the global memory, all interpolation must be done *in-kernel*, which increases both the number of instructions per integration step, and the amount of memory required by the kernels. By delegating the interpolation process to this dedicated hardware, we are able to reduce both the size and the computational complexity of the kernels. The size is especially important in this case, as smaller kernels allow for a higher degree of parallelism.

While we expect the use of texture-filtering interpolation to be beneficial for the running time of our algorithm (which we will demonstrate in the next Section), we do identify one possible trade-off. One property of in-kernel interpolation is that the values of the eight surrounding voxels can be stored in either the local memory of the thread, or in the shared memory of the multiprocessor. Doing so increases the size of the threads, but also allows them to reuse some of this data, thus reducing the memory throughput requirements. Using texture-filtering interpolation, we cannot store the surrounding voxel values, so we need to read them again in every integration step. Thus, in-kernel interpolation may require a significantly lower memory throughput than texture-filtering interpolation, especially for small step sizes (in which case it takes multiple steps for a fiber to cross a cell). We analyze this trade-off through experimentation in the next section.

## 5. Experiments and Results

For the experiments presented in this section, we used a synthetic data set of 1024 × 64 × 64 voxels with 2048 predefined seed points. The seed points were distributed randomly to mimic the low spatial coherence of the fibers, and their initial directions were chosen in such a way that no thread would terminate prematurely due to its fiber leaving the volume (i.e., all fibers stay within the volume for the predefined maximum number of integration steps, running parallel to the long edge of the volume). While neither the shape and content of the volume nor the fact that no fibers leave the volume prematurely can be considered realistic, this does allow us to benchmark the algorithm under maximum load.

All experiments were conducted on an NVIDIA GTX 260, a mid-range model with 24 multiprocessors (for a total of 192 scalar processors) and 1 GigaByte of device memory [35].

It should be noted that we only consider the actual fiber tracking process for our benchmarks. The data preparation stage (which includes preprocessing and inverting the tensors, and computing the derivatives of the inverse tensors) has also been implemented in CUDA, but is considered outside of the scope of this paper due to its low complexity, trivial parallelization, and low running times compared to the tracking stage. On the GTX 260, the data preparation stage requires roughly 50 milliseconds per million voxels [36], and only needs to be executed once per image. The overhead resulting from the communication between the CPU and GPU lies in the order of a few milliseconds.

### 5.1. Texture Filtering versus In-Kernel Filtering

**Figure 6:**
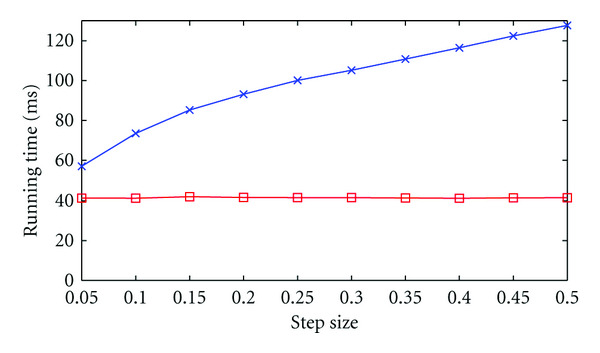
Running time for varying step size for in-kernel interpolation (blue) and texture-filtering interpolation (red).

In Section 4.4, we stated that using the dedicated texture filtering hardware for the trilinear interpolation step of our algorithm would significantly reduce the size and complexity of our kernel, allowing for an increase in performance. We also identified a possible trade-off: for small step sizes, in-kernel interpolation might be faster than texture-filtering interpolation, as the former allows for data reuse, while the latter does not. We test this hypothesis by varying the step size between 0.05 (i.e., twenty steps per cell on average) and 0.5 (two steps). The measured running times for the two proposed interpolation methods are shown in Figure 6. From this, we can conclude that, while small step sizes do indeed reduce the running times for in-kernel interpolation, texture-filtering interpolation is still the faster option for typical step size values (between 0.1 and 0.2).

### 5.2. Limiting Factor

The performance of CUDA programs is usually limited either by the maximum *memory throughput* between the device memory and the multiprocessors, or by the maximum *computational throughput* of the scalar processors. To find out which of these two is the limiting factor for our algorithm, we first compute its performance in terms of memory throughput and computational throughput.

- When computing 2048 fibers, each executing 4096 integration steps, the algorithm takes approximately 165 milliseconds to complete. For each integration step, a single thread needs to read 8 voxels ∗ 4 tensors ∗ 6 unique tensor elements ∗ 4 bytes  =  768 bytes, and it writes 12 bytes (3D coordinates of the new point). This gives us a total memory transfer of 780 ∗ 2048 ∗ 4096 *≈* 6.54 GB. Dividing this by the running time, we get an effective memory throughput of approximately 3.97 GB/s, which is well below the maximum 111.9 GB/s of the GTX 260.
- To compute the computation throughput in FLOPS (floating-point operations per second), we first decompile the CUDA program using the decuda software. By inspecting the resulting code, we learn that each integration step uses 256 floating-point instructions. Note that this does not include any instructions needed for interpolation, since we are using the dedicated texture filtering hardware for this purpose. The algorithm performs a total of 256 ∗ 2048 ∗ 4096 = 2,147,483,648 floating-point operations, giving us a computational throughput of roughly 13 GFLOPS. Again, this is well below the specified maximum of the GTX 260, which is 715 GFLOPS.

Since neither the memory throughput nor the computational throughput is close to the specified maximum, we need to determine the limiting factor in a different way. We first rule out the computational throughput as the limiting factor by replacing the current second-order Runge-Kutta (RK2) ODE solver by a simple Euler solver. This does not reduce the amount of data transferred between device memory and multiprocessors, but it does reduce the number of floating-point operations per integration step from 256 to 195. This, however, does not significantly impact the running time (165.133 ms for RK2 versus 165.347 ms for Euler), indicating that the computational throughput (i.e., processor load) is not a limiting factor in our case.

To prove that the memory throughput is indeed the limiting factor, we need to reduce the amount of data read in each integration step, without changing the number of instructions. We do so by using the knowledge that the four input tensors of our synthetic data set share a number of duplicate values. By removing some of the redundant reads, we can reduce the memory requirements of each thread, without compromising the mathematical correctness of the algorithm. The results of this experiment are listed in Table 1. From this, we can conclude that the performance of our algorithm is limited by the memory throughput, despite the actual throughput being significantly lower than the theoretical maximum of the GTX 260. Possible explanations for this discrepancy are listed in Section 6.

### 5.3. Speed-Up Factor

#### 5.3.1. Setup

To evaluate the performance of our CUDA implementation, we compare its running times to those of a C++ implementation of the same algorithm running on a modern CPU. In order to fully explore the performance gain, we use four different CUDA-supported GPUs: the Quadro FX 770M, the GeForce 8800 GT, the GeForce GTX 260, and the GeForce GTX 470. The important specifications of these GPUs are shown in Table 2. It should be noted that our CUDA implementation was developed for the GTX 260 and was not modified for execution on the other GPUs. In particular, this means that we did not optimize our algorithm to make use of the improved CUDA features of the more recent GTX 470 GPU.

The CPU running the C++ implementation is an Intel Core i5 750, which has four cores, with a clock speed of 2.67 GHz. In terms of both date of release and price segment, the i5 750 (released in fall 2009) is closest to the GTX 470 (spring 2010); at the time of writing, both are considered mid- to high-range consumer products of the previous generation.

For this benchmark, we use a brain DTI image with dimensions of 128 × 128 × 30 voxels. Seed points are placed in a small, two-dimensional region of 22 by 4 voxels, located in a part of the corpus callosum. An approximation of the seed region, as well as the resulting fibers, is shown in Figure 8. Seed points are randomly placed within this region, with a random initial direction. The number of seed points varies from 1024 to 4096. This test thus closely mimics a real-life application of the ray-tracing algorithm, as demonstrated in Figure 8.

#### 5.3.2. CPU Implementation

**Figure 7:**
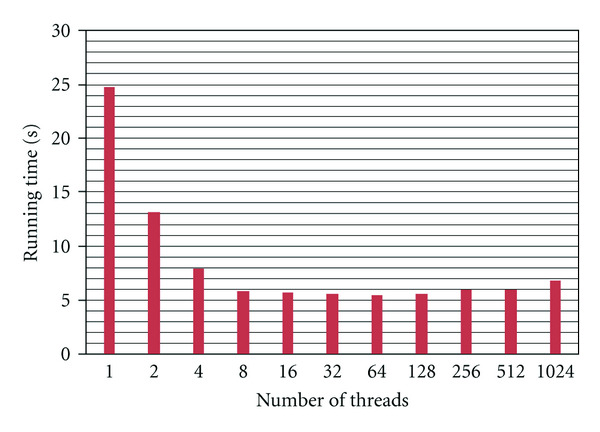
Running times (in seconds) for the multithreaded CPU implementation of our algorithm.

**Figure 8:**
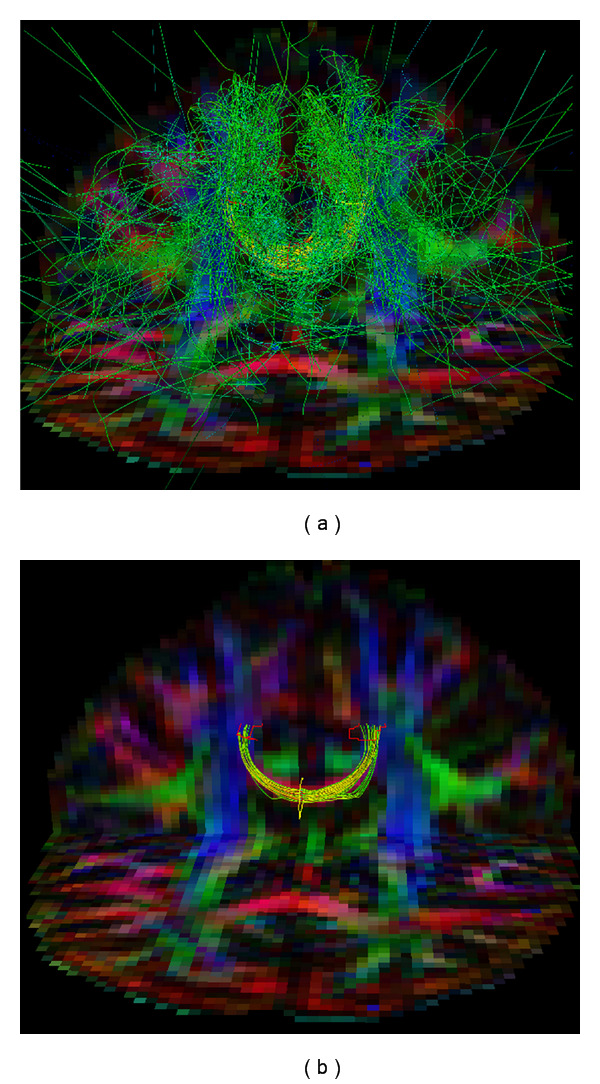
Fibers computed using the CUDA ray-tracing algorithm in real data. (a) All fibers computed from a seeding region in part of the corpus callosum. (b) In order to properly analyze the computed fibers, a postprocessing step is needed. In this case, fibers were filtered through two target regions of interest and ranked and colored according to their Connectivity Measure value (see Section 2.3). The yellow polygon approximates the seeding region used for the benchmarks in Section 5.3. Both images were generated in the DTITool [11].

Our C++ implementation of the algorithm features support for multiple threads of execution (multithreading), which allows it to utilize the parallelism offered by the CPU's four cores. Let *S* be the number of seed points and *N* the number of threads. We assign *S*/*N* seed points to each CPU thread, thus having each thread compute *S*/*N* fibers. We have measured the running times of this CPU implementation for several different values of *N*, and with *S* set to 4096 points. The results of this benchmark can be seen in Figure 7. From these results, we can conclude that parallelizing the CPU implementation (using essentially the same fiber-level parallelism as for our GPU implementation) can reduce the running times by a factor of roughly 4.5. The fact that the performance increases for *N* larger than the number of cores can be attributed to the fact that a CPU core can switch to a different thread when the active thread is waiting for data from the main memory of the PC. From Figure 7, we can also conclude that 64 threads is the best configuration for this algorithm and this CPU.

#### 5.3.3. GPU Benchmark Results

We performed the same benchmarking experiment on the four CUDA-enabled GPUs, and we compared the running times to those of the best CPU configuration. The results for this test are shown in Table 3. From the results, we can conclude that our CUDA implementation significantly reduces the running time of the ray-tracing algorithm. Even on a mid-range GPU for laptops like the FX 770M, the running time is reduced by a factor of roughly two times. Using a high-end, recent GPU like the GTX 470, we are even able to achieve a speed-up factor of 40 times, which greatly increases the applicability of the algorithm. The differences in speed-up factors between the GPUs can be explained in part by the differences in bandwidth—which was identified as the limiting factor in Section 5.2—and by the number of processors.

## 6. Discussion

In previous sections, we described a CUDA implementation of a geodesic fiber tracking method. This CUDA program uses the knowledge that fibers can be computed independently of one another to parallelize these computations, thus reducing running times by a factor of up to 40 times, compared to multithreaded CPU implementations of the same algorithm.

While the algorithm does allow for meaningful parallelization, we do note two problems that make full exploitation of the parallelism offered by the GPU challenging.

- The algorithm requires a large amount of internal storage; between the four input tensors, the Christoffel symbol, and the position and direction of the fiber, even the most efficient version of our implementation still required 43 registers per thread, while typical values for CUDA algorithms are between 8 and 32.
- More importantly, the algorithm requires a large amount of data transfer, and as a result, the memory throughput becomes its limiting factor. This problem is far from trivial to solve, and while some options do exist, they all have their own downsides.
  (1) Reducing the number of bits per input value would lower the required memory throughput, but comes at the cost of reduced accuracy.
  (2) Allowing fibers to reuse the input data used for interpolation greatly increases the size of the kernel and does not work with texture-filtering interpolation, the use of which has been shown to be very beneficial in our case.
  (3) Sharing the input data between threads in one multiprocessor requires some sort of spatial coherence between the fibers, which is extremely difficult to guarantee.

As noted, the memory throughput between the device memory and the multiprocessors is the limiting factor for our performance. However, as noted in Section 5.2, the actual throughput is well below the theoretical maximum of the device. Below, we list some possible causes for this difference.

(iii) Most Random-Access Memory (RAM) configurations experience a certain overhead when subsequent reads access different parts of a data range. Since a low spatial locality of the seed points will lead to such scattered access pattern, this overhead may explain our relatively low memory throughput.
(iv) According to CUDA documentation [32], texture reads have been optimized for 2D spatial locality, presumably using a space-filling curve. The absence of spatial locality prevents our algorithm from utilizing these optimizations.
(v) The throughput of the texture fetching and filtering units may become a limiting factor when large numbers of voxels are involved. The documentation of the GTX 260 states that it should be able to process 36.9 billion texels per second [33], while our implementation only loads 39 billion bytes (of multibyte texels) per second. However, this figure is based on 2D bilinear interpolation, while we use 3D trilinear interpolation.

We expect the first point to be the main contributing factor, though we have not conducted experiments to either prove or disprove this hypothesis.

The GPU-based acceleration of the geodesic ray-tracing algorithm for DTI is a useful technique, but its implementation poses several challenges. Part of the problem in accelerating numerical integration algorithms such as the one under discussion in the paper is that it almost inevitably introduces unpredictable memory access patterns, while existing CUDA algorithms generally use access patterns that are more regular and predictable, and thus easier to optimize. This is a fundamental problem without an easy solution, and one that is not restricted to CUDA-enabled GPUs, but applies to other parallel platforms as well. Still, despite our implementation achieving suboptimal performance, we do believe that its significant speed-up factor of up to 40 times, coupled with the low cost and high availability of CUDA-enabled hardware, makes it a practical solution to the computational complexity of the geodesic ray-tracing algorithm for fiber tracking, and a good starting point for future acceleration of similar algorithms.

## 7. Conclusion and Future Work

In this paper, we discussed the GPU-based acceleration of a geodesic ray-tracing algorithm for fiber tracking for DTI. One of the advantages of this algorithm is its ability to find multivalued solutions, that is, multiple possible connections between regions of the white matter. However, the high computational complexity of the algorithm, combined with the fact that we need to compute a large amount of trajectories if we want to find the right connection, makes it slow compared to similar algorithms. To overcome this problem, we accelerated the algorithm by implementing a highly parallel version on a GPU, using NVIDIA's CUDA platform. We showed that despite the large kernel size and high memory requirements of this GPU implementation, we were able to speed up the algorithm by a factor of up to 40. This significant reduction in running time using cheap and widely available hardware greatly increases the applicability of the algorithm.

Aside from further optimization of our current GPU implementation, with the aim of further reducing its running time, we identify two possible extensions of the work presented in this paper.

- At the moment, the computed fibers are downloaded back to the CPU, where they are postprocessed and subsequently visualized. A more direct approach would be to directly visualize the fibers computed by our algorithm, using the available rendering features of the GPU. If we can also implement the necessary postprocessing steps in CUDA, we can significantly reduce the amount of memory that needs to be transferred between the CPU and the GPU, thus accelerating the complete fiber tracking pipeline.
- The Region-to-Region Connectivity method is a valuable application of the geodesic ray-tracing algorithm. This method introduces three extra steps: (1) computing a suitable Connectivity Measure, either while tracking the fibers or during a post-processing step, (2) filtering the computed fibers through a target region, and (3) sorting the remaining fibers according to their Connectivity Measure, showing only those fibers with high CM values. These three steps have currently been implemented on a CPU, but implementing them on a GPU can reduce the overall processing time of the fiber tracking pipeline, as noted above.
- The DTI model is limited in its accuracy by its inability to model crossing fibers. High Angular Resolution Diffusion Imaging (HARDI) aims to overcome this limitation by measuring and modeling the diffusion in more directions [37]. An extension of the algorithm presented in Section 2.3 which uses HARDI data rather than DTI data would theoretically be more accurate, particularly in complex areas of the white matter. Such an extension may, for example, be realized by using the Finsler metric tensor (which depends both on the position and local orientation of the fiber), rather than the Riemannian metric tensor [38, 39]. While the extended algorithm for HARDI would likely be more complex in terms of both number of computations and amount of required memory, a CUDA-based acceleration using the parallelization principles presented in this paper will still be significantly faster than any CPU implementation.

We note that, while these extensions would be valuable additions, the current CUDA implementation already provides a practical and scalable solution for the acceleration of geodesic ray-tracing.

## Figures and Tables

**Table 1:** Effects on the running time and total memory throughput of changing the amount of data read per voxel.

Data/Step (Byte)	Time (ms)	Bandwidth (GB/s)
768	165.133	38.8
640	134.124	40.0
512	103.143	41.6
384	90.554	35.6

**Table 2:** Specifications of the GPUs in the benchmark test of Section 5.3. Source: http://www.nvidia.com/content/global/global.php.

	Device memory (MB)	Memory bandwidth (GB/s)	Number of scalar processors
Quadro FX 770M	512	25.6	32
GeForce 880 GT	512	57.6	112
GeForce GTX 267	896	111.9	192
GeForce GTX 470	1280	133.9	448

**Table 3:** Benchmark results for GPU and CPU implementation of the geodesic ray-tracing algorithm. For each configuration, we show the running time (T) in seconds, and the Speed-Up factor (SU) relative to the best CPU timing, see Figure 7.

	CPU	FX 770M		8800 GT		GTX 260		GTX 470	
Number of seeds	*T *	* T *	SU	*T *	SU	*T *	SU	*T *	SU
1024	1.403	0.761	1.8	0.273	5.1	0.225	6.2	0.087	16.1
2048	2.788	1.388	2.0	0.448	6.2	0.244	11.4	0.093	30.0
3072	4.185	1.995	2.1	0.760	5.5	0.256	16.3	0.107	39.1
4096	5.571	2.772	2.0	0.900	6.2	0.301	18.5	0.139	40.0
